# The inSIGHT study: costs and effects of routine hysteroscopy prior to a first IVF treatment cycle. A randomised controlled trial

**DOI:** 10.1186/1472-6874-12-22

**Published:** 2012-08-08

**Authors:** Janine G Smit, Jenneke C Kasius, Marinus JC Eijkemans, Carolien AM Koks, Ron Van Golde, Jurjen GE Oosterhuis, Annemiek W Nap, Gabrielle J Scheffer, Petra AP Manger, Annemiek Hoek, Mesrure Kaplan, Dick BC Schoot, Arne M van Heusden, Walter KH Kuchenbecker, Denise AM Perquin, Kathrin Fleischer, Eugenie M Kaaijk, Alexander Sluijmer, Jaap Friederich, Joop SE Laven, Marcel van Hooff, Leonie A Louwe, Janet Kwee, Jantien J Boomgaard, Corry H de Koning, Ineke CAH Janssen, Femke Mol, Ben WJ Mol, Helen L Torrance, Frank JM Broekmans

**Affiliations:** 1 Department of Reproductive Medicine and Gynaecology, University Medical Center Utrecht, Utrecht, The Netherlands; 2 University Medical Center Utrecht, Julius Center for Health Sciences and Primary care, Utrecht, The Netherlands; 3 Department of Obstetrics and Gynaecology, Maxima Medical Center, Veldhoven, The Netherlands; 4 Department of Obstetrics and Gynaecology, Maastricht University Medical Center, University of Maastricht, Maastricht, The Netherlands; 5 Department of Obstetrics and Gynaecology, Medical Spectrum Twente, Enschede, The Netherlands; 6 Department of Obstetrics and Gynaecology, Rijnstate Hospital, Arnhem, The Netherlands; 7 Department of Obstetrics and Gynaecology, Gelre Hospital, Apeldoorn, The Netherlands; 8 Department of Obstetrics and Gynaecology, Diakonessen Hospital Utrecht, Utrecht, The Netherlands; 9 Department of Obstetrics and Gynaecology, University Medical Center Groningen, University of Groningen, Groningen, The Netherlands; 10 Department of Obstetrics and Gynaecology, Ropcke-Zweers hospital, Hardenberg, The Netherlands; 11 Department of Obstetrics and Gynaecology, Catharina Hospital, Eindhoven, The Netherlands; 12 Department of Obstetrics and Gynaecology, Antonius Hospital, Nieuwegein, The Netherlands; 13 Department of Obstetrics and Gynaecology, Isala Clinics, Zwolle, The Netherlands; 14 Department of Obstetrics and Gynaecology, Medical Center Leeuwarden, Leeuwarden, The Netherlands; 15 Department of Obstetrics and Gynaecology, University Medical Center St Radboud, Nijmegen, The Netherlands; 16 Department of Obstetrics and Gynaecology, Onze Lieve Vrouwe Gasthuis, Amsterdam, The Netherlands; 17 Department of Obstetrics and Gynaecology, Wilhelmina Hospital, Assen, The Netherlands; 18 Department of Obstetrics and Gynaecology, Gemini Hospital, Den Helder, The Netherlands; 19 Department of Obstetrics and Gynaecology, Division of Reproductive Medicine, Erasmus MC, Rotterdam, The Netherlands; 20 Department of Obstetrics and Gynaecology, Sint Franciscus Gasthuis, Rotterdam, The Netherlands; 21 Department of Gynaecology and Reproductive Medicine, Leids University Medical Center, University of Leiden, Leiden, The Netherlands; 22 Department of Obstetrics and Gynaecology, Westfriesgasthuis, Hoorn, The Netherlands; 23 Department of Obstetrics and Gynaecology, Tergooi hospitals, Blaricum, The Netherlands; 24 Department of Obstetrics and Gynaecology, Groene Hart Hospital, Gouda, The Netherlands; 25 Department of Obstetrics and Gynaecology, Centre for Reproductive Medicine, Academic Medical Centre, Amsterdam, The Netherlands; 26 Department of Obstetrics and Gynaecology, Academic Medical Center, University of Amsterdam, Amsterdam, The Netherlands

**Keywords:** Hysteroscopy, Subfertility, IVF

## Abstract

**Background:**

In in vitro fertilization (IVF) and intracytoplasmatic sperm injection (ICSI) treatment a large drop is present between embryo transfer and occurrence of pregnancy. The implantation rate per embryo transferred is only 30%. Studies have shown that minor intrauterine abnormalities can be found in 11–45% of infertile women with a normal transvaginal sonography or hysterosalpingography. Two randomised controlled trials have indicated that detection and treatment of these abnormalities by office hysteroscopy after two failed IVF cycles leads to a 9–13% increase in pregnancy rate. Therefore, screening of all infertile women for intracavitary pathology prior to the start of IVF/ICSI is increasingly advocated. In absence of a scientific basis for such a policy, this study will assess the effects and costs of screening for and treatment of unsuspected intrauterine abnormalities by routine office hysteroscopy, with or without saline infusion sonography (SIS), prior to a first IVF/ICSI cycle.

**Methods/design:**

Multicenter randomised controlled trial in asymptomatic subfertile women, indicated for a first IVF/ICSI treatment cycle, with normal findings at transvaginal sonography. Women with recurrent miscarriages, prior hysteroscopy treatment and intermenstrual blood loss will not be included. Participants will be randomised for a routine fertility work-up with additional (SIS and) hysteroscopy with on-the-spot-treatment of predefined intrauterine abnormalities versus the regular fertility work-up without additional diagnostic tests. The primary study outcome is the cumulative ongoing pregnancy rate resulting in live birth achieved within 18 months of IVF/ICSI treatment after randomisation. Secondary study outcome parameters are the cumulative implantation rate; cumulative miscarriage rate; patient preference and patient tolerance of a SIS and hysteroscopy procedure. All data will be analysed according to the intention-to-treat principle, using univariate and multivariate logistic regression and cox regression. Cost-effectiveness analysis will be performed to evaluate the costs of the additional tests as routine procedure. In total 700 patients will be included in this study.

**Discussion:**

The results of this study will help to clarify the significance of hysteroscopy prior to IVF treatment.

**Trial registration:**

NCT01242852

## Background

Despite the numerous advances in the field of in vitro fertilisation (IVF) and intracytoplasmic sperm injection (ICSI), the maximum implantation rate per embryo transferred is still approximately 30%. Even if both ovum pick-up and fertilization occur successfully in the process of IVF, there is a large unexplained gap between successful embryo transfer and occurrence of pregnancy. Implantation failure presents a major clinical challenge and is a cause of considerable stress to patients and clinicians in assisted reproductive technology (ART). Besides the psychological and physical burden of each IVF treatment cycle, it also adds to the considerable costs associated with fertility treatment [1]. If progress is to be made in improving implantation rates, a greater understanding of the factors which determine successful implantation is required.

Implantation failure could be due to the embryo, uterine environment or a combination of both. Even minor uterine cavity abnormalities, such as endometrial polyps, small submucous myomas, adhesions, and septa are considered to have a negative impact on the chance to conceive through IVF [2]. The prevalence of unsuspected intrauterine abnormalities, diagnosed by hysteroscopy prior to IVF, has been reported to be 11–45% [3-13]. Therefore, it has been proposed that these abnormalities should be diagnosed and treated in order to optimize the condition of the uterine environment and thus the outcome of IVF treatment. However, this recommendation is not based on high quality evidence [3,5,7-10]. In addition, the benefits of hysteroscopy in patients who will undergo a first IVF/ICSI treatment have not yet been investigated.

At present, the basic work-up for evaluation of the uterine cavity prior to IVF consists of transvaginal ultrasound, possibly followed by saline infusion sonography (SIS), hysterosalpingography (HSG) or hysteroscopy. The accuracy of HSG in assessment of the uterine cavity integrity in subfertile patients has been reported to be rather disappointing [14,15]. SIS is increasingly considered to be useful in diagnosing intrauterine abnormalities. It is an inexpensive, non-invasive diagnostic test, and has been proven to be very accurate [16,17]. Yet hysteroscopy is still considered to be the gold standard. It has become easy to perform in an outpatient clinic without anesthesia. Moreover, hysteroscopy enables diagnosis and treatment of intrauterine pathology in the same setting.

The NVOG (Dutch society of Obstetrics and Gynaecology) as well as the ESHRE (European Society for Human Reproduction and Embryology) and RCOG (Royal College of Obstetricians and Gynaecologists) do not recommend SIS nor hysteroscopy as initial investigation prior to starting IVF [18-20]. It has been argued that the significance of treating unsuspected intrauterine abnormalities has not yet been proven.

So far, none of the guidelines considered the most recent literature on this topic. In a retrospective cohort analysis, Gera et al. compared the pregnancy rate after operative hysteroscopy of patients with intrauterine abnormalities at SIS to the pregnancy rate of patients with a normal uterine cavity. A 31.6% increase in pregnancy rate was observed after treatment of detected abnormalities [21]. Furthermore, two randomized trials reported exceptional improvements in pregnancy rates after office hysteroscopy and instant treatment of detected pathology in patients after two failed IVF attempts. Intervention resulted in a 9–13% increase in clinical pregnancy rate in the subsequent IVF cycle [7,9]. These results endorsed the findings of other, previously published prospective studies [3,5]. Despite some methodological weaknesses in the study design, the results of these studies indicate a trend towards a beneficial effect of screening hysteroscopy on IVF outcome. This finding, combined with the observed high prevalence of intrauterine abnormalities, has led to a general debate on the beneficial effect of pre-IVF work-up of the uterine cavity.

The current study aims to clarify the additive value of routine SIS and/or hysteroscopy prior to IVF/ICSI . Also, patient preferences and the cost-effectiveness of these tests as routine procedures for assessment of the uterine cavity prior to starting IVF will be assessed.

## Methods

### Study design

The inSIGHT trial is a multicenter randomised controlled trail (RCT). A total of twenty-five academic and non-academic centers in the Netherlands will participate. An economic analysis is incorporated to assess the cost-effectiveness of the investigated tests as routine procedures prior to IVF. Inclusion was started in May 2011.

This study will be conducted according to the principles of the Declaration of Helsinki and in accordance with the Medical Research Involving Human Subjects Act (WMO). The study protocol has been approved by the medical ethical review committee of the University Medical Center Utrecht (MEC 10–272) and by the board of directors of all participating centers. The protocol is registered at clinicaltrial.gov NCT01242852.

### Recruitment, consent and randomisation

**Figure 1:**
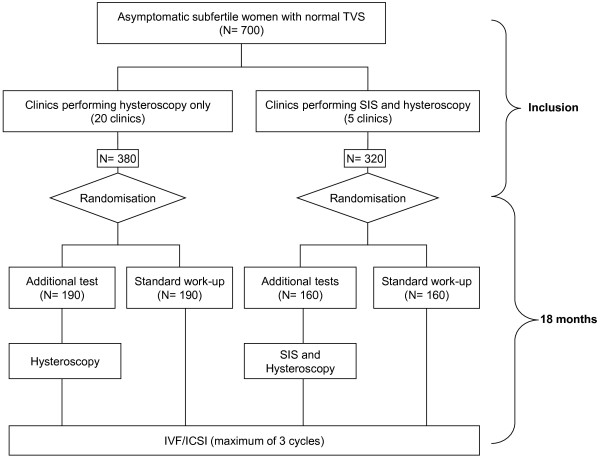
Flowchart inSIGHT study.

Women with an indication for a first IVF/ICSI treatment cycle will be informed about this trial by their attending physician. They will also receive written patient information. One to two weeks after the first visit, the coordinating investigator will counsel the woman. If a woman agrees to participate in the study, informed consent will be obtained and randomisation will be performed for one of two strategies: routine infertility work-up followed by immediate IVF (control arm) or the routine work-up with additional diagnostic tests ((SIS and) hysteroscopy) with on-the-spot treatment of intrauterine pathology followed by IVF (intervention arm) (Figure 1). Randomisation will be performed centrally by a web-based randomisation program and will be stratified according to recruiting center.

### Study population

All women with primary or secondary infertility scheduled for a first IVF/ICSI treatment cycle at one of the participating hospitals who have a normal transvaginal ultrasound are considered to be eligible for inclusion. A normal transvaginal ultrasound is defined as no visible intracavitary pathology (e.g. submucous myomas, polyps or septa) and will be performed in the follicular phase of the menstrual cycle. Intramural myomas without impression or deformation of the uterine cavity are incorporated in the definition of a normal ultrasound. Exclusion criteria are recurrent miscarriage, defined as 2 or more miscarriages prior to the 20^th^ week of gestation, prior hysteroscopy treatment and intermenstrual blood loss.

### Interventions

#### Intervention arm

All women in the intervention group will undergo routine fertility work-up with additional tests. In five of the twenty-five research hospitals the additional tests consist of SIS and hysteroscopy. In all the other participating hospitals the additional test will consist of solely a hysteroscopy.

SIS will be performed prior to the hysteroscopy on CD 3–9. A speculum will be inserted vaginally and up to 20 ml of sterile saline solution will be infused into the uterine cavity through a catheter to distend the endometrial cavity. A transvaginal transducer will be used to scan the uterine cavity. The findings at SIS will be recorded in a standardised manner on the case record form (CRF).

The hysteroscopy examination will be scheduled in the early-mid follicular phase of a menstrual cycle (day 3–12). The hysteroscopy examinator will be blinded for SIS outcomes. A vaginoscopic approach will be used without anesthesia in an outpatient setting. Only if the examination cannot be accomplished due to patient intolerance, the possibility of a paracervical block will be offered. Hysteroscopy will be performed using a 5-mm outer-diameter continuous flow hysteroscope with a 5 French working channel and a 30 direction of view. The uterine cavity will be distended by saline infusion. The endocervical canal, uterine cavity, tubal orifices and endometrium will be inspected methodically and findings recorded on a standardized form. Intrauterine abnormalities will be defined as endometrial polyps, septate uterus, adhesions and chronic or acute endometritis. A sample of the endometrium will be obtained for histological examination by a gentle biopsy with a ‘pipelle’ biopsy canulla or grasping forceps. Therapeutic hysteroscopy will be performed in the same hysteroscopy session if any of the predefined intrauterine abnormalities is detected. It is left to the judgement of the operator physician to decide to perform a septum resection in a subsequent session with laparoscopic observation. After the hysteroscopy, the resected tissue will be examined histologically by a pathologist for the presence of chronic or acute endometritis.

To assess patient tolerance of SIS and a diagnostic/therapeutic hysteroscopy, all patients will be asked to complete a standardized questionnaire, containing questions about the level of tolerance and acceptability of both procedures.

#### Control arm

All women allocated to the control group will be scheduled for IVF or ICSI and undergo standard treatment, without SIS or hysteroscopy.

#### Fertility treatment

All randomised women, be it allocated to intervention or control arm, will undergo IVF. In the intervention group IVF treatment will be started within 3 months after hysteroscopy. Rec-FSH or HMG combined with a GnRH agonist protocol (Leuprolide or Triptorelin 100 microgram) or a GnRH antagonist protocol (Ganirelix/Cetrorelix 0.25 mg/d) will be used. Final oocyte maturation will be achieved by the administration of HCG when 3 or more follicles of more than 16 mm are present. Oocyte retrieval will be carried out 36 hours after HCG administration. Luteal phase supplementation consists of 600 mg natural micronised progesterone in three separate dosages (Urogestan®/Progestan® 100 mg 3x2/day) starting in the evening after oocyte retrieval and continued until 18 days after ovum pick-up.

### Withdrawal of individual patients

Subjects can abandon the study at any time for any reason if they wish to do so without any consequences. The investigator can decide to withdraw a subject from the study for urgent medical reasons.

## Outcome measures

The primary endpoint of this study will be the cumulative ongoing pregnancy rate resulting in live birth achieved within 18 months of IVF/ICSI treatment after randomisation.

Secondary study parameters/endpoints will be the cumulative implantation and miscarriage rate within 18 months of IVF/ICSI treatment after randomisation, cost calculations of SIS, hysteroscopy procedures and the IVF treatment, patient preference and tolerance of SIS and diagnostic/therapeutic hysteroscopy procedure and presence of unexpected intracavitary abnormalities (e.g. endometrial polyps, septate uterus, adhesions and endometritis) prior to IVF/ICSI.

To investigate the cost-effectiveness of SIS and hysteroscopy as routine procedures prior to IVF treatment, a cost analysis will be performed. The economic evaluation is performed from a societal perspective. Differentiation will be made between direct medical costs (all health care sector costs), direct non-medical costs (costs outside the health care sector that are affected by health status or health care) and indirect costs of the fertility treatment (costs of sick leave due to fertility treatment). For medical costs, the process of care is divided into three cost stages (cost of SIS, cost of hysteroscopy, cost of IVF treatment), which can reoccur in case of repeat treatment cycles. Costs will be computed from the period of inclusion to the end of follow-up (18 months).

Health care utilisation in the fertility treatment consists of visits to hospital, ultrasound, gonadotrophins, oocyte retrieval, lab work of IVF (including various laboratory tests) and hospital care. Volumes of health care resources use will be measured prospectively alongside the clinical study. At this stage, direct non-medical and indirect costs may be generated if women are absent from paid work, either for visiting the fertility clinic during IVF treatment, or for sick leave associated with physical or psychological side-effects of this treatment. The Health and Labor Questionnaire [22] will be used to document absence from paid work.

## Statistical analysis

### Sample size calculation

Based on the literature, the increase in ongoing pregnancy in women with recurrent IVF failure after treatment of predefined abnormalities of the uterine cavity is found to be 9–32% in the subsequent IVF/ICSI cycles [5,7,9].

In a group of women indicated for a first IVF/ICSI treatment cycle, the difference in cumulative live birth rate after 18 months of IVF/ICSI treatment is estimated to be 10% between the patients with and without hysteroscopy (40% versus 30%). The number of patients needed to have 80% power (with alpha = 0.05) to detect such a difference is 350 per study arm.

To be able to answer the question on the benefit of using SIS as a pre-selection tool the following sample size calculation for patients needing to have both SIS and hysteroscopy was made. From previous literature, the prevalence of unsuspected intrauterine abnormalities is considered to be 12% and the sensitivity of SIS compared to hysteroscopy to be 95% [11]. SIS and hysteroscopy need to be performed in 160 women to achieve a 95% confidence interval of 10% (85–100%). This means that a total of 320 women need to be randomised in the five hospitals in this study that perform SIS.

### Economic analysis

For each strategy, live birth rate as well as the average costs per patient will be estimated and used to calculate cost-effectiveness ratios. Incremental cost-effectiveness ratios will be estimated for two strategies of additional diagnostic/therapeutic procedures: SIS followed by hysteroscopy if SIS is abnormal or no SIS but direct hysteroscopy. These two strategies will be compared to no additional investigations (routine fertility investigation followed directly by IVF/ICSI). Robustness of the results (costs and health outcomes) for various assumptions and parameters estimates will be explored in sensitivity analyses and visualized in ICER-graphs and cost-effectiveness acceptability curves.

### Statistic analysis

SPSS and Excel will be used to perform the statistical analysis. A probability less than 0.05 will be considered to be significant. Data will be presented for the two arms of the study group. Data will be expressed as means +/− standard deviation and proportions or rates. The analysis will be by intention to treat.

Descriptive analysis will be used to assess the prevalence of predefined minor intrauterine abnormalities at SIS and office hysteroscopy. Comparisons between the two arms of the randomised group will be done by applying Chi-square testing to crude rates of cumulative implantation and ongoing pregnancy and by using life table analysis to account for the factor of time to implantation or pregnancy. The difference in tolerability of SIS will be compared to the tolerability of diagnostic and therapeutic hysteroscopy making use of the Student’ *t*-test analysis of the VAS score.

The relative contribution of predefined cavitary abnormalities versus other predictive factors like female age and duration of infertility will be assessed by univariate and multivariate logistic regression and Cox regression.

## Discussion

Despite the great advances in the field of IVF/ICSI, implantation failure is still high. This not only causes considerable stress to the woman but is also associated with high costs of the fertility treatment. The uterine cavity is considered to play an important role in successful implantation. Even minor abnormalities, such as endometrial polyps, submucous myomas, adhesions or septa, are thought to impair the chance to conceive [2]. Studies have shown a high prevalence of unsuspected intrauterine abnormalities prior to IVF [3-12,23]. In addition, two RCTs reported improvement in pregnancy rates after hysteroscopy in the subsequent IVF cycle in patients with two previous failed IVF attempts. Currently, hysteroscopy is not recommended as routine investigation in the fertility work-up because of a lack of robust evidence. The present inSIGHT study is a large randomised controlled trial in which the effects on pregnancy rates and costs of SIS and hysteroscopy will be studied. The results of this study, which are expected in 2015, will help to clarify the significance of hysteroscopy prior to IVF/ICSI treatment.

## Abbreviations

IVF, In vitro fertilisation; ICSI, Intracytoplasmic sperm injection; SIS, Saline infusion sonography; HSG, Hysterosalpingography; NVOG, Nederlandse Vereniging voor Obstetrie en Gynaecologie; ESHRE, European Society for Human Reproduction and Embryology; RCOG, Royal College of Obstetricians and Gynaecologists; RCT, Randomised controlled trial; CRF, Case Record Form.

## Competing interests

The authors declare that they have no competing interests.

## Authors’ contribution

JS is responsible for the overall logistical aspects of the trial and drafted the manuscript. JK, HT and FB have contributed to the development of the protocol and study design. JK applied for a grant. FB and HT have overall responsibility for the trial. CK, RvG, GE, AN, GS, PM, AH, MK, DS, AvH, WK, DP, KF, EK, AS, JF, JL, MH, LL, JK, JB, CK, CJ, FM and BWM are responsible for implementation of the study and inclusion of eligible patients. All authors read and approved the final manuscript.

## Pre-publication history

The pre-publication history for this paper can be accessed here:

http://www.biomedcentral.com/1472-6874/12/22/prepub
